# Real-world evaluation of supportive care using an electronic health record text-mining tool: G-CSF use in breast cancer patients

**DOI:** 10.1007/s00520-022-07343-5

**Published:** 2022-08-31

**Authors:** Sylvia A. van Laar, Kim B. Gombert-Handoko, Sophie Wassenaar, Judith R. Kroep, Henk-Jan Guchelaar, Juliette Zwaveling

**Affiliations:** 1 Department of Clinical Pharmacy and Toxicology, Leiden University Medical Center, Albinusdreef 2, 2333ZA Leiden, The Netherlands; 2 Department of Medical Oncology, Leiden University Medical Center, Leiden, The Netherlands

**Keywords:** Granulocyte-colony stimulating factor, Chemotherapy-induced febrile neutropenia, Breast cancer, Clinical practice pattern, Text-mining

## Abstract

**Purpose:**

Chemotherapy-induced febrile neutropenia (FN) is a life-threatening and chemotherapy dose-limiting adverse event. FN can be prevented with granulocyte-colony stimulating factors (G-CSFs). Guidelines recommend primary G-CSF use for patients receiving either high (> 20%) FN risk (HR) chemotherapy, or intermediate (10–20%) FN risk (IR) chemotherapy if the overall risk with additional patient-related risk factors exceeds 20%. In this study, we applied an EHR text-mining tool for real-world G-CSF treatment evaluation in breast cancer patients.

**Methods:**

Breast cancer patients receiving IR or HR chemotherapy treatments between January 2015 and February 2021 at LUMC, the Netherlands, were included. We retrospectively collected data from EHR with a text-mining tool and assessed G-CSF use, risk factors, and the FN and neutropenia (grades 3–4) and incidence.

**Results:**

A total of 190 female patients were included, who received 77 HR and 113 IR treatments. In 88.3% of the HR regimens, G-CSF was administered; 7.3% of these patients developed FN vs. 33.3% without G-CSF. Although most IR regimen patients had ≥ 2 risk factors, only 4% received G-CSF, of which none developed neutropenia. However, without G-CSF, 11.9% developed FN and 31.2% severe neutropenia.

**Conclusions:**

Our text-mining study shows high G-CSF use among HR regimen patients, and low use among IR regimen patients, although most had ≥ 2 risk factors. Therefore, current practice is not completely in accordance with the guidelines. This shows the need for increased awareness and clarity regarding risk factors. Also, text-mining can effectively be implemented for the evaluation of patient care.

**Supplementary Information:**

The online version contains supplementary material available at 10.1007/s00520-022-07343-5.

## Introduction

Quantifying healthcare outcomes in clinical practice is a crucial step towards the improvement of cancer patients’ care [1]. The electronic health record (EHR) is a valuable source of real-world medical data, including, e.g., demographics, vital signs, laboratory data, and medication orders, which can be used for treatment evaluation [2, 3]. However, most of the information is stored in unstructured text, specifically regarding treatment outcomes, e.g., in pathology reports, and detailed adverse drug events in narrative notes [4, 5]. Manual data extraction has been the standard extraction method for EHR data, which is known to be time-consuming and error-prone [3, 6]. Novel natural language processing and text-mining techniques facilitate automatized data extraction from EHR [6–8] and therefore enable the evaluation of treatments and guidelines in clinical practice.

For years, granulocyte colony-stimulating factors (G-CSF) are used to prevent chemotherapy-induced neutropenia in breast cancer patients [9]. Neutropenia is one of the most serious and common adverse events of myelosuppressive chemotherapy [10, 11]. Complications of neutropenia are fever, or febrile neutropenia (FN), due to opportunistic infections, which often require intravenous antibiotic treatment and hospitalization [12–14]. Moreover, patients developing severe FN often receive chemotherapy dose reductions or treatment delays, which is associated with worse survival outcomes [13].

Prophylactic use of granulocyte colony-stimulating factors (G-CSF), e.g., filgrastim, has shown to reduce the severity and duration of neutropenia and the incidence of FN by 50–90% [10, 14, 15]. G-CSF use can also result in adverse events, mainly mild to moderate bone pain (25–36%), but also potentially secondary myeloid neoplasms [16]. Furthermore, broad use of G-CSF was previously assumed to be a significant financial burden to the healthcare system; the cost-effectiveness of G-CSF is highly related with both the FN risk and the G-CSF costs in clinical practice [17, 18].

FN incidence is primarily related to the type and intensity of the chemotherapy regimen. Therefore, European Organisation for Research and Treatment of Cancer (EORTC) and National Comprehensive Cancer Network (NCCN) guidelines indicate that primary prevention of FN with G-CSF (PP G-CSF) should be applied if patients receive treatment with a high-risk (HR) (> 20%) for FN. For patients receiving a chemotherapy regimen with an intermediate FN risk (IR) of 10–20%, other patient-related risk factors should be considered to define if the overall FN risk exceeds 20%, and thus, PP G-CSF is indicated [10, 19, 20]. Although multiple patient-related risk factors are related with an increased FN risk, e.g., age ≥ 65 years, advanced disease, and female gender, the exact risk attribution of these factors to FN is not yet defined [10]. This may complicate decision-making as to whether or not to administer PP G-CSF. It is already shown that in clinical practice, not all patients receive PP G-CSF when recommended [11, 21]. However, real-world evidence on PP G-CSF utilization linked to risk factors for FN is limited.

Even though several guidelines on G-CSF use for clinical practice are present, it is not clear to what extend these are followed. Therefore, the aim of this study is to retrospectively review guideline adherence for HR and IR chemotherapy regimens in a breast cancer population by collecting data from the EHR with text mining.

## Methods

### Patient population

All patients aged 18 years and older with breast cancer were included if they started a HR or IR chemotherapy regimen between January 2015 and February 2021 at Leiden University Medical Center (LUMC), Leiden, the Netherlands. Patients participating in the DIRECT study were excluded, since these patients by study design were not allowed to receive PP G-CSF treatment and therefore might bias results [22]. The study protocol was reviewed and approved by the Medical Ethics Review Committee of the LUMC, Leiden, who waived the need for informed consent.

### Data collection method

We performed the data collection from the EHR with rule-based text-mining software (CTcue B.V., Amsterdam, the Netherlands). This tool enables extraction of structured (e.g., laboratory results and medical prescriptions) and unstructured (free-text notes) data and the immediate conversion of results into a dataset. For unstructured notes, it enables to search for (combinations of) keywords and shows all notes matching with these results. We validated the software previously for data extraction to evaluate metastatic renal cell carcinoma treatments, which showed high accuracy (> 90%) for data collection from structured data [23]. All used queries for patient inclusion and data collection are in supplementary file 1. All used queries for patient inclusion and data collection are available in supplementary file 1. Patients were identified by a combination of the selected chemotherapy treatments in their medication history, mention of the treatment regimen in the notes, and a diagnosis treatment code for breast cancer. All patients and their regimens were manually validated within the software tool. Additionally, for data extracted from unstructured text (tumor receptor characteristics, type of treatment, G-CSF use and incidence of (febrile) neutropenia), we also performed manual validation of the data by reviewing the identified within the tool. G-CSF use and incidence of (febrile) neutropenia were further manually validated by EHR review.

### Patient-, disease- and treatment characteristics

All risk factors from the EORTC guideline that could be evaluated in retrospect were included in this study. These were the following patient- and disease characteristics with specified cutoff values per risk factor (RF): age (RF: age > 65 years), sex (RF: female gender), length and weight (RF: body surface area < 2.0 m^2^), hemoglobin (RF: hemoglobin < 12 g/dl), ALT (RF: abnormal liver transaminases | ALT > 35 U/l), AST (RF: abnormal liver transaminases | AST > 30 U/l), eGFR (RF: renal disease | eGFR < 60 ml/min/1.73m^2^), absolute neutrophil count (RF: low pretreatment ANC |< 2 × 10^9^ cells/L[24]), serum albumin (RF: albumin < 3.5 g/dl), performance status (RF: performance status > 0), previous treatments (RF: prior chemotherapy), and treatment type (curative or palliative, RF: advanced disease/metastasis) [10].

We did not include the following risk factors: prior episodes of FN, since we estimated the risk prior to cycle one; antibiotic prophylaxis, since patients in The Netherlands do not receive antibiotic prophylaxis; and cardiovascular disease, one or more comorbidities, and prior infections, since the high variability in free-text terminology combined with the uncertainty whether these risk factors are noted structurally in the EHR would result in an incomplete, and potentially incorrect risk estimation.

### Outcome measurements

The primary outcome was the incidence of FN from chemotherapy initiation until 21 days after last treatment cycle. FN is defined as the ANC < 0.5 × 10^9^ cells/L, or ANC < 1.0 × 10^9^ cells/L, predicted to fall below 0.5 × 10^9^ cells/L within 48 h, with fever or clinical signs of sepsis. Fever is defined as rise in axillary temperature > 38.5 °C for 1 h [10]. Also, the incidence of grade 3 or higher neutropenia was collected, which is defined as ANC < 1.0 × 10^9^ cells/L (Common Terminology Criteria for Adverse Events v.5.0). We included cases of FN and neutropenia if they met the definition based on structured data, or when noted in unstructured text by a treating physician.

### Statistical analysis

Data management and analysis was performed using R 4.1.0 (R CoreTeam, 2021). Descriptive statistics were used to describe patient, treatment, and disease characteristics. The number of patients on HR or IR chemotherapy regimen receiving PP G-CSF that developed neutropenia or FN was summarized in percentages and visualized in a Sankey plot. With the Student’s *t*-test, the number of risk factors between the subgroups was compared. Also, chi-square tests or Fisher’s exact test if expected frequency was lower than five, was performed to compare risk factors between subgroups.

## Results

In total, 190 breast cancer patients, which received an IR or HR regimen between January 2015 and February 2021, were included. All patients were female and had a median age of 52.6 years (± 11.4 years). Baseline patient, tumor, and treatment characteristics are shown in Table 1.

**Table 1:** Patient, tumor, and treatment characteristics

	HR regimen patients *n* = 77 Median (*1st–3rd quartile) or n* (%)	IR regimen patients *n* = 113 Median *(1st–3rd quartile*) or *n* (%)
Patient characteristics		
Female gender	77 (100)	113 (100)
Age (years)	51 (38–57)	55 (48–64)
Body surface area (m2)	1.81 (1.72–1.92)	1.85 (1.72–1.92)
Hemoglobin (U/l)	8.5 (8–8.8)	8.4 (8.0–8.8)
Absolute neutrophil count (U/l)	4.1 (3.5–5.5)	4.5 (3.5–5.8)
Albumin (g/dl)	46 (44–48)	45 (44–48)
ALT (U/l)	20 (15–26.3)	20 (15–26.5)
AST (U/l)	21 (19–25)	22 (18–26.25)
eGFR (ml/min/1.73m2)	89 (81–90)	90 (78–90)
WHO performance status		
0	41 (53.2)	49 (43.4)
1	3 (3.9)	19 (16.8)
2	1 (1.3)	2 (1.8)
* Missing*	32 (41.6)	43 (38.1)
Tumor receptor characteristics		
Progesterone receptor positive	34 (44.2)	69 (61.1)
Estrogen receptor positive	42 (55.2)	89 (78.8)
HER2 receptor positive	1 (1.3)	12 (10.6)
* Missing*	1 (1.3)	4 (3.5)
Treatment characteristics		
Previous chemotherapy treatment	4 (5.2)	16 (14.2)
Type of treatment		
Neo-adjuvant	49 (63.6)	32 (28.3)
Adjuvant	25 (32.5)	67 (59.3)
Palliative	1 (1.3)	6 (5.3)
* Missing*	2 (2.6)	8 (7.0)

*IR*, intermediate-risk; *HR,* high-risk; *ALT*, alanine transaminase; *AST*, aspartate transaminase; *eGFR*, estimated glomerular filtration rate; *HER2*, human epidermal growth factor receptor 2

Table 2 presents an overview of the included chemotherapy regimens. In total, patients received 77 HR and 113 IR chemotherapy regimens. Most applied HR regimens were dose dense doxorubicin and cyclophosphamide (ddAC) → paclitaxel and carboplatin (32.5%), ddAC → paclitaxel (31.2%), and the combination of paclitaxel, doxorubicin, and cyclophosphamide (TAC, 24.7%). Most applied IR regimens were a combination of doxorubicin and cyclophosphamide (AC, 52.2%) and AC → docetaxel (25.7%).

**Table 2:** Included high- and intermediate-risk chemotherapy treatment regimens

	Number of patients per treatment *n* = 190 *n* (%)
High-risk treatments	77 (40.5)
ddAC	3 (3.9)
ddAC → docetaxel	4 (5.2)
ddAC → paclitaxel	24 (31.2)
ddAC → paclitaxel and trastuzumab	1 (1.3)
ddAC → paclitaxel and carboplatin	25 (32.5)
Paclitaxel → ddAC	1 (1.3)
TAC	19 (24.7)
Intermediate-risk treatments	113 (59.5)
AC	59 (52.2)
AC → docetaxel	29 (25.7)
Cyclophosphamide	2 (1.8)
Docetaxel	4 (3.5)
Docetaxel and cyclophosphamide	7 (6.2)
FEC	1 (0.9)
FEC, trastuzumab and pertuzumab	5 (4.4)
FEC → docetaxel	7 (6.2)

*AC*, doxorubicin and cyclophosphamide; *ddAC*, dose dense AC; *FEC*, 5-fluorouracil, epirubicin, and cyclophosphamide; *TAC*, docetaxel, doxorubicin and cyclophosphamide

**Fig. 1:**
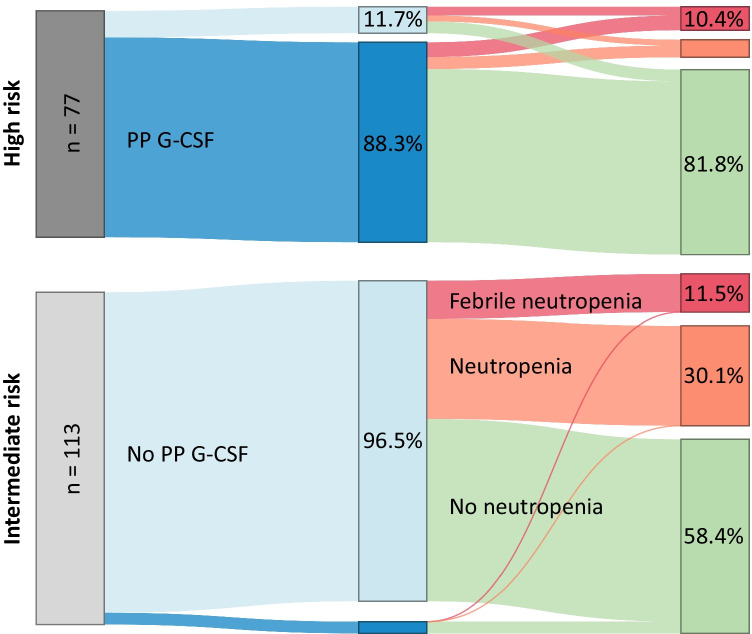
Proportion of patients that received primary granulocyte-colony stimulating factor (PP G-CSF) treatment and developed neutropenia (≥ grade 3) or febrile neutropenia stratified for intermediate- and high-risk chemotherapy treatments

Table 3 summarizes the proportion of patients that received PP G-CSF, and developed neutropenia, and FN, stratified per risk group. These results are visualized in Fig. 1. Overall, in 37.9% of chemotherapy regimens, PP G-CSF was administered at the start of the treatment regimen, 88.3% during HR treatments and 3.5% during IR regimens. The incidence of severe (≥ grade 3) neutropenia in the overall cohort was 21.1%; 11.1% of the patients developed FN at least once. The incidence of neutropenia and FN combined was higher in patients treated with IR regimens (41.6%) than in HR-treated patients (18.2%). However, FN incidence in both groups was comparable and around 10%. In the HR treatment group, 33.3% of the patients who did not receive PP G-CSF developed FN, in contrast to 7.3% of the patients who received PP G-CSF. Furthermore, in the IR treatment group, none of the four patients that started with PP G-CSF developed FN and 11.9% in the group who did not receive PP G-CSF.

**Table 3:** Proportion of patients with high and intermediate risk regimens who received primary prophylaxis using granulocyte colony-stimulating factor (PP G-CSF) from the start of treatment and developed severe (≥ grade 3) neutropenia or febrile neutropenia (FN)

	PP G-CSF	Febrile neutropenia	Neutropenia	No neutropenia	Total
High-risk regimen	Yes, *n* (%)	5 (7.3)	4 (5.9)	59 (86.8)	68 (100)
	No, *n* (%)	3 (33.3)	2 (22.2)	4 (44.4)	9 (100)
	Subtotal, *n* (%)	8 (10.4)	6 (7.8)	63 (81.8)	77 (100)
Intermediate-risk regimen	Yes, *n* (%)	0 (0)	0 (0)	4 (100)	4 (100)
	No, *n* (%)	13 (11.9)	34 (31.2)	62 (56.9)	109 (100)
	Subtotal, *n*	13 (11.5)	34 (30.1)	66 (58.4)	113 (100)
	Total, *n* (%)	21 (11.1)	40 (21.1)	129 (67.9)	190 (100)

**Fig. 2:**
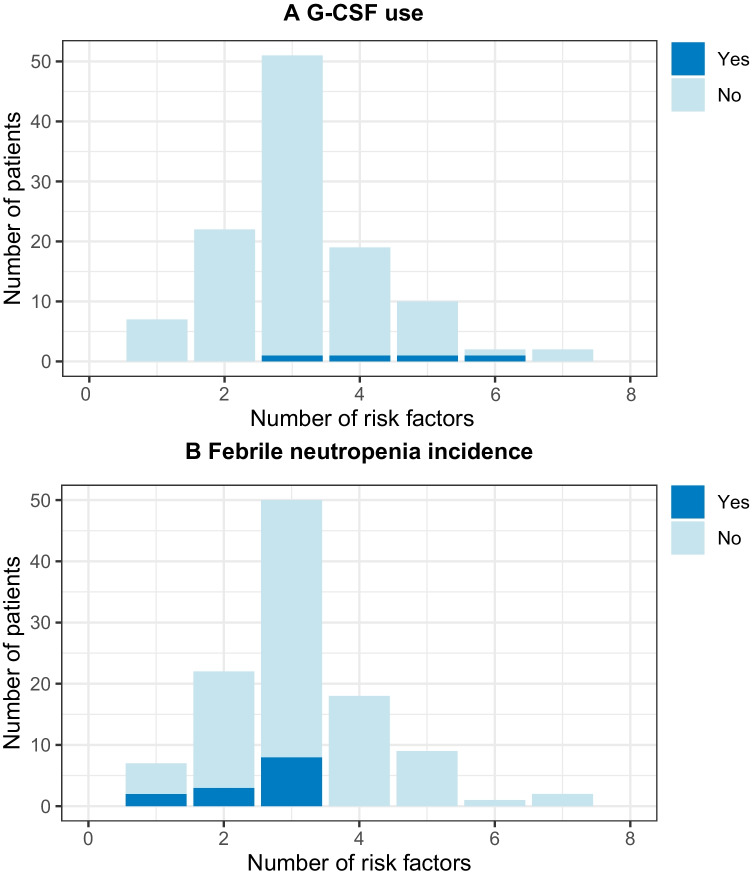
Number of confirmed risk factors per patient in the intermediate risk group stratified by G-CSF use (**A**), and patients not receiving PP G-CSF by FN status (**B**)

We analyzed the presence of additional patient-related risk factors in the IR-treated group. A mean of 3.4 risk factors per patient was found, ranging from one to eight risk factors per patient (Fig. 2A). Patients who received PP G-CSF (*n* = 4) had a mean of 5 risk factors, and without PP G-CSF (*n* = 109) 3.3 (*p* = 0.13)*.* No significant difference was found between the distribution of individual risk factors between both groups (Table 4).

**Table 4:** Risk factors present in the intermediate risk group stratified by PP G-CSF use, and patients not receiving PP G-CSF by FN status

	A. PP G-CSF use			B. Febrile neutropenia		
Risk factor	Yes *n* = 4 *n* (%)	No *n* = 109 *n* (%)	*p*-value	Yes *n* = 13 *n* (%)	No *n* = 96 *n* (%)	*p*-value
Female gender	4 (100)	109 (100)	-	13 (100)	96 (100)	^-^
Age > 65 years	2 (50)	20 (18.3)	0.17	0 (0)	20 (20.8)	0.060
Body surface area < 2 m2	4 (100)	94 (86.2)	0.58	9 (69.2)	85 (89.5)	0.041
*Missing*	0	1		0	1	
Hemoglobin < 12 g/dl	2 (50.0)	11 (10.1)	0.065	1 (9.1)	10 (9.1)	0.61
Absolute neutrophil count ≤ 5.2 × 10^9/l	2 (50.0)	65 (65.6)	0.44	7 (53.8)	58 (67.4)	0.34
*Missing*	0	10		0	10	
Albumin < 3.5 g/dl	0 (0)	2 (2.1)	0.92	0 (0)	2 (2.3)	0.61
*Missing*	0	12		2	10	
Liver function: ALT ≥ 35 U/l or AST ≥ 30 U/l	2 (50)	20 (18.5)	0.17	2 (15.4)	18 (18.9)	0.55
*Missing*	0	1		0	1	
Kidney function: eGFR < 60 ml/min/1.73m2	0 (0)	3 (2.8)	1	0 (0)	3 (3.2)	0.67
*Missing*	0	3		0	3	
WHO performance status > 0	1 (33.3)	20 (29.9)	0.66	1 (11.1)	19 (32.8)	0.18
*Missing*	1	42		4	38	
Previous treatment	2 (50)	14 (12.8)	0.095	0 (0)	14 (14.6)	0.14
Palliative treatment	1 (25.0)	5 (5.0)	0.21	0 (0)	5 (5.5)	0.55
*Missing*	0	8		2	6	

*ALT*, alanine transferase; *AST*, aspartate transferase; *eGFR*, estimated glomerular filtration rate. *p*-value < 0.05 was defined as significant

Of the patients on an IR regimen that did not receive PP G-CSF, thirteen (11.9%) developed FN (Table 3). Figure 2B shows that these patients had a lower mean of risk factors versus the patients who did not develop FN (2.5 versus 3.4; *p* = 0.0044). Furthermore, the risk factor BSA below 2 m^2^ was more prevalent in the group who did not develop FN (Table 4).

## Discussion

In this study, we investigated whether EHR text mining could be applied to evaluate G-CSF use among breast cancer patients in clinical practice. The high recall rate both and the results of the within-program manual validation indicate that adherence to guidelines can be performed using EHR text mining. We found that, in general, G-CSFs were not as often administered as primary prophylaxis as indicated in the EORTC guideline. Not all patients (8.8%) who received HR regimens started with G-CSF prophylaxis. Also, even though almost all patients with IR regimens had two or more of the investigated risk factors, only 4% received PP G-CSF. This resulted in an overall neutropenia incidence of 32%, including 11% FN. Our results indicate that prophylactic treatment with G-CSF should be optimized to further prevent the occurrence of (febrile) neutropenia.

### High-risk chemotherapy treatment

Despite guidelines indicating 100% of the patients on a HR regimen should receive PP G-CSF, we report a PP G-CSF use of 88%. An undertreatment of the HR population was also found by Gawade et al., who reported 76.4% PP G-CSF use [21]. Also, PP G-CSF use over time seems to be improved over the recent years [25]. In the HR regimen patients that received PP G-CSF according to the guidelines, only 7% still developed FN, which is comparable to the incidence of FN (9.5%) found in breast cancer patients receiving PP G-CSF (intention-to-treat) for 5 days by Clemons et al. [26]. However, approximately a third of the patients without prophylaxis developed FN. This difference may be directly related to the lack of primary FN prophylaxis; however, numbers in this group are small. In our hospital, most of the omissions of PP G-CSF in the HR group were unintentional. This is potentially related to the fact that G-CSFs are not prescribed and ordered as regular medication in the EHR for hospitalized patients. In the future, these errors could be prevented by incorporating G-CSF prescription into the predefined HR chemotherapy treatment protocol in the EHR.

### Intermediate-risk chemotherapy treatment

Almost none (4%) of the patients treated with an IR chemotherapy received PP G-CSF. As PP G-CSF should only be administered if the cumulative FN risk per individual patient exceeds 20%, the risk estimation of patient receiving an IR treatment should be based on additional risk factors [10]. The low use of G-CSF in this population is remarkable as all patients have at least one of the investigated risk factors, female gender, and most have more than two, which may indicate standard G-CSF prescription for these patients. In comparison to the 4% of PP G-CSF use in our study, both Gawade et al. and Bacrie et al. reported around 18% of PP G-CSF use in IR risk patients [21, 27]. Even though the low use of G-CSFs in this population, the FN incidence was moderate (12%) and a substantial amount of the patients developed grades 3–4 neutropenia (31%). Higher G-CSF use could further have prevented cases of severe neutropenia, which can also result in chemotherapy dose delay or reductions [28, 29].

None of the investigated risk factors was significantly related to PP G-CSF prescriptions in our patients. It is remarkable that 20 patients (18%) aged > 65 years did not receive PP G-CSF, since this is the most prominent mentioned risk factor in the guidelines [10]. However, none of the risk factors was significantly associated with the incidence of FN in the group of patients who did not receive PP G-CSF. On the contrary, more risk factors were found in the group without FN, and low BSA specifically was more present in this group. Therefore, even though the small sample sizes, it is questionable to what extent the investigated risk factors substantially contribute to FN development and G-CSF prescription in this IR group. To assess whether dose reductions were applied as alternative preventative measure, we assessed dose reductions on the initial dose and found that approximately 5% of patients started at a dose of 70–80% of the standard starting dose. Thus, in our population, dose reductions play only a minor role or not at all in the risk for FN. Also, antibiotic prophylaxis of FN is not used in this population. Therefore, overall, it may have been other, more difficult to assess factors that could have contributed more to both decision-making for PP G-CSF use and real-world FN risk, e.g., risk factors as radiation therapy and the excluded risk factors [12].

Gawade et al., who compared mainly comorbidities to PP G-CSF initiation by retrospectively reviewing a large medical claims database, also showed that risk factors seemed not to influence PP G-CSF initiation in the IR group, although this was suggested in the NCCN guidelines [21]. Furthermore, Lyman et al. compared the model-predicted and physician-predicted FN risk and showed a weak correlation [30]. This underlines that there may be a difference in how physicians weigh patient-, disease- and treatment-related risk factors; therefore, they suggest the need for continuous education on FN risk factors, G-CSF toxicity, guidelines, and appropriate PP G-CSF use. Zooming in, we note that the underlying problem might be the broad definition of some risk factors and their contribution to clinical outcomes. Therefore, besides continuous education, we recommend, firstly, clarification of the risk factors that play a major role in chemotherapy-induced FN, and, secondly, clearer and simple guidelines which state how these risk factors should be weighed. Therefore, the development of a scoring system, comparable to the system developed to estimate the FN risk in patients who receive low-risk treatments, could also be beneficial for the intermediate- and high-risk group [31].

Moreover, G-CSFs were known to be costly and as a consequence, the choice for treatment above > 20% FN risk only was highly related with the cost-effectiveness [15]. However, new biosimilars are proven to be significantly cost-saving and thereby lower the threshold for the application of G-CSF and simplification of the guidelines [32, 33]. This could not only further lower the FN risk, but also the risk on severe neutropenia. Nonetheless, G-CSF can result in adverse events, and potential benefits of G-CSF use should always outweigh risks.

In this study we applied EHR text-mining software. This enabled fast, structured, and pseudonymized patient inclusion and data extraction. Using this method, missing data of the included risk factors, in general, was limited*.* However, not all potential risk factors could (fully) be assessed retrospectively. Partially, as the EHR is a secondary source and not all patient data are equally well documented in the EHR, e.g., in this study, we could only report the performance status for approximately 60% of the patients. But also, to some extent, because definitions were unclear or too broad, e.g., one or more comorbidities. We still performed manual validation of the critical end-points G-CSF use and FN incidence after text mining, since these were mainly documented in unstructured text. Since only a selection of the data had to be validated, this process was faster compared to complete manual data extraction. Our study shows that a text-mining tool can be an effective method to review adherence to guidelines and that results can be used as a concrete starting point to optimize patient care.

## Conclusion

By application of text mining to the EHR, we were able to review G-CSF use in daily practice in breast cancer patients. PP G-CSF use among HR regimen patients was high, however not maximal, and undertreatment resulted in a higher incidence of FN. Most IR regimen patients had more than two risk factors and were therefore entitled to the use of PP G-CSF. However, few received PP G-CSF which could have prevented the occurrence of FN and neutropenia. Therefore, current practice is not completely in accordance with the guidelines, in particular for patients treated with IR regimens, and may result in unnecessary toxicity for patients. We conclude that awareness of risk factors related with neutropenia should be enlarged and these risk factors could be more clearly defined in the guidelines. Finally, our study shows that text-mining methods can be effectively implemented to review daily practice for the evaluation and improvement of patient care.

## Supplementary Information

Below is the link to the electronic supplementary material.

Supplementary file1 (XLSX 22 KB) Supplementary file 1: Queries for patient selection and data extraction in CTcue

## Data Availability

The data that support the findings of this study are available from the corresponding author upon reasonable request.

## Code availability

Used queries are summarized in supplementary file 1 and available upon reasonable request.
